# Multidisciplinary diagnosis and treatment training simulation by paired teachers using case-based teaching of oral histopathology that promotes clinical thinking

**DOI:** 10.1038/s41598-023-28786-8

**Published:** 2023-01-27

**Authors:** Shan Wang, Yanfen Qiu

**Affiliations:** 1 Department of Oral Pathology, School of Stomatology, Hainan Medical College, Haikou, 571199 People’s Republic of China; 2 Department of Stomatology, The Second Affiliated Hospital of Hainan Medical University, Haikou, 570216 People’s Republic of China; 3 Department of Oral Radiology, Hospital of Stomatology, The First Affiliated Hospital, Harbin Medical University, Harbin, 150001 People’s Republic of China

**Keywords:** Health care, Medical research

## Abstract

The multidisciplinary diagnosis and treatment (MDT) model has significant advantages in the diagnosing and treatment of intricate cases. In addition, it can eliminate subspecialty barriers and provide a more accurate and individualized diagnosis and treatment plan. It has been assumed that the future development of diagnosis and treatment will retain this course, and the MDT is an essential type of clinical thinking, especially for medical students. This study attempted to guide stomatology undergraduates’ thinking via an MDT simulation in oral histopathology practicums through typical case-based education and paired-teachers’ explanations. The aim was to cultivate clinical thinking among students based on individual cases and to improve class participation and students’ clinical thinking ability. Forty-six undergraduates in a 5-year stomatology program who enrolled in 2018 participated in a simulation MDT model. Ten typical clinical cases were selected, and they were previously collected and analyzed by clinicians in accordance with the simulation MDT model and handed out to teachers and students before the class. Two to three cases were interpreted by teacher pairs that included a pathologist (oral pathology teacher) and a radiologist (oral imaging teacher). The rest of the cases were used for simulation MDT student groups in class. The oral pathology teacher and oral imaging teacher illustrated the corresponding data from typical cases in advance. The simulation MDT group members acting as a surgeon, pathologist, or radiologist demonstrated their own cases assigned randomly before the class. A curriculum satisfaction survey illustrated that the simulation MDT group agreed that simulation MDT was novel for them, and they had a strong sense of participation. The mimic MDT training with typical cases guided by paired teachers was useful for establishing student clinical thinking.

## Introduction

A multidisciplinary diagnosis and treatment (MDT) team is a working group including experts from different clinical and laboratory fields. The team deals with diseases to establish the best treatment plan for patients through regular consultation and then to implement it via related disciplines or a combination of multiple disciplines^1–3^.

During the mid-nineteenth century, the invention of the microscope and the progress in histopathology that followed enhanced the interaction between pathologists, physicians, and surgeons based on cytological evidence-based medicine, thereby greatly promoting clinical multidisciplinary collaboration^4,5^. In China, MDT inpatient services are provided in approximately 38% of class 3A hospitals that implement the MDT model. By contrast, approximately 94% of class 3A hospitals in the United States that implement the MDT model offer MDT inpatient services to improve efficiency and quality of care^6^. Therefore, effective MDT implementation provides a sufficient basis for personalized treatment and is promising for future diagnosing and treatment in China. For stomatology undergraduates, early introduction to and thinking about MDT are very important for cultivating the capacity for comprehensive diagnosis and treatment in China.

An oral or maxillofacial neoplasm is a common disease of the oral and maxillofacial region, and it includes cysts, tumors, tumorlike lesions, and non-neoplastic diseases^7,8^. These diseases show complex nosology and diverse biological features. Therefore, oral and maxillofacial surgeons face higher requirements for preoperative diagnosis and intraoperative histopathology. In past oral histopathology courses at our college, teachers of oral histopathology generally listed the diseases required in the teaching program and explained their microscopic features. Then, they let students observe slides under the microscope in the internship course. Undergraduates noted that cysts, tumors, or tumorlike pathological changes were obscure and complex, although teachers of oral histopathology tried to present the learning materials as clearly as possible. Most undergraduates would mechanically repeat what the book said to pass the final exam. As an old Chinese saying goes, “we understand only—and approximately—the how, not the why.” While entering clinical practice and encountering patients, students cannot fully understand the original instructional materials about neoplasms and thus face barriers in clinical thinking. For this reason, the authors of this paper attempted to guide the students to establish a “simulation MDT” students’ group in a practicum on oral histopathology in the form of case-based teaching combined with cooperative teaching (paired teachers).

The term “case-based teaching”^9–14^ denotes the teaching of some representative cases of oral maxillofacial neoplasms encountered in the author’s clinical work. Case-based learning has been validated as an optimal method for educating undergraduate dental students. Furthermore, clinical data may contribute to diagnostic imaging, especially regarding lesions with multiple differential diagnoses^15^. Therefore, teacher pairs included one teacher of oral imaging and one of oral pathology. The authors of this paper hoped that the above innovation would be a better approach to the cultivation of clinical thinking in students based on individual cases and will improve the students’ class participation.

## Methods and method

### Teaching process

**Figure 1:**
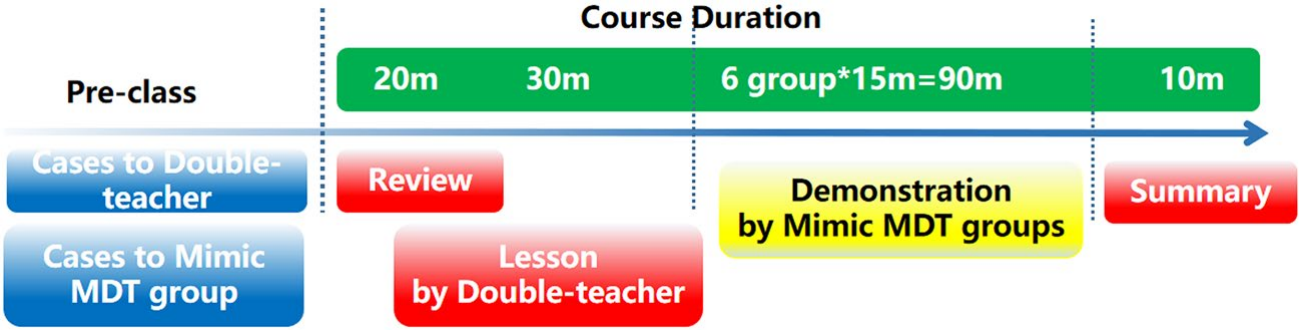
Course time schedule.

The teaching process included the following three modules: (1) Preparation of typical cases; (2) “Teacher pairs” + “Case-based teaching” (an oral imaging teacher and oral pathology teacher explain together the typical cases with special clinical characteristics); (3) a simulation MDT group illustrating the cases. See Fig. 1 for details.

1. Preparation of typical cases: Ten typical clinical cases that were collected and analyzed regularly by clinicians in accordance with the simulation MDT model were selected.

Two to three cases were interpreted by teacher pairs; the rest of the cases were used for simulation MDT student groups.

**Figure 2:**
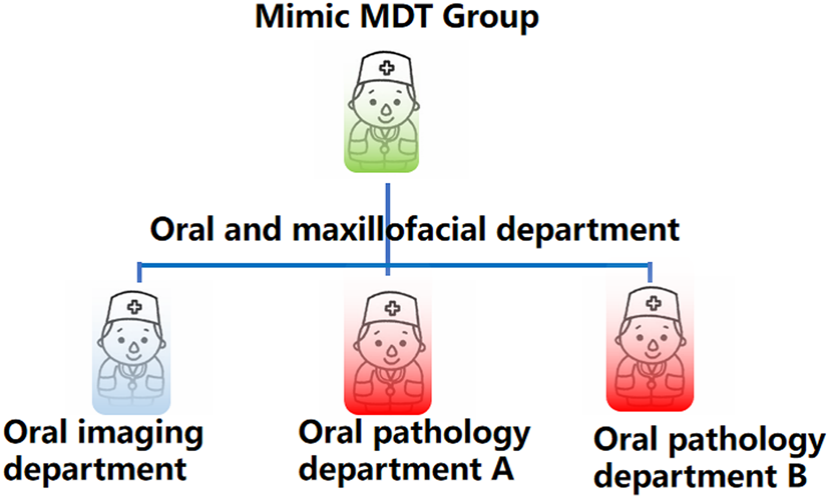
Grouping in the Mimic MDT training including a oral surgeon,a oral radiologist and one or two oral pathologist. Photoshop CS5 software is used to generate cartoon drawing in Fig. 2(ADOBE * PHOTOSHOP * CS5 EXTENDED12.0 × 32 version http://www.adobe.com/cn).

(2) “Teacher pairs” + “case-based teaching”: The oral pathology teacher reviewed the theoretical knowledge on oral and maxillofacial neoplasms for 20 min; the oral imaging teacher and oral pathology teacher illustrated the corresponding data from typical cases in 30 min; the simulation MDT groups respectively demonstrated for 90 min their own cases randomly assigned before the class.
(3) Simulation MDT group presentations: The undergraduates of a 5-year stomatology program who participated in this study as of 2018 were enrolled in the School of Stomatology, ***Medical University (a total of 46 students). Ethical approval for this study was obtained from the Ethics Committee of the First Affiliated Hospital of ***Medical University. All methods were performed in accordance with the relevant guidelines and regulations. Informed consent was obtained from all subjects involved in this study. There were 24 students in Group A and 22 students in Group B, and the groups were to be taught separately. Students were assigned randomly to groups according to the number assigned when they entered the school, which was of great help for creating a harmonious teaching atmosphere and ecological environment and establishing interaction between teachers and students thanks to an appropriate teacher–student ratio. Students in Groups A and B received the same educational means and teaching knowledge on the account of considering the principle of equity in education. The cases were to be worked through twice. Each group consisted of three to four members who played respectively the roles of an oral radiologist, an oral and maxillofacial surgeon, and one or two oral pathologists. Each group reported for 15 min. See Fig. 2 for details.
(4) The teachers summarized the performance of the mimic MDT groups in 10 min.

### Training in the process of thinking ability

**Figure 3:**
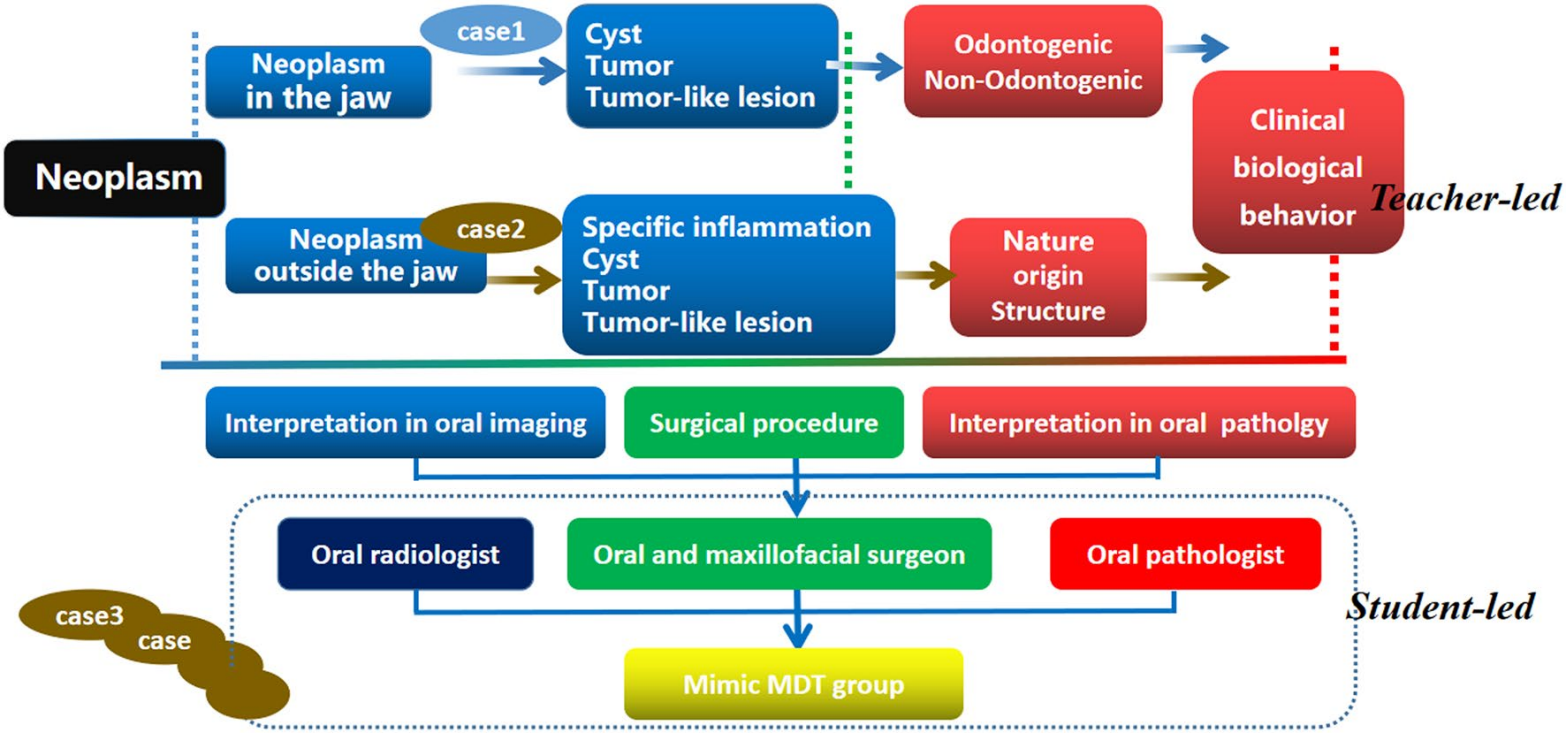
Cultivation mode of clinical thinking ability of Mimic MDT guided by case teaching combined with paired-teachers.

1. *General information on the cases for teacher illustration (Case 1 as an example)* Case 1 was a neoplasm in the jawbone. The oral pathology teacher introduced clinical information including symptoms, medical history, and physical examination findings and guided the students on how to practice clinical thinking. Potential diseases could be maxillofacial cysts, tumors, tumorlike lesions, or metastatic tumors.
2. *Illustration from an oral radiologist (oral-imaging teacher)* The oral-imaging teacher explained the cone beam computed tomography, 3D reconstruction, and X-ray imaging characteristics of Case 1. Because students who participated in this study learned medical imaging during the first semester of their junior year but lacked systematic imaging coursework associated with the oral-cavity field, the oral-imaging teacher emphasized the addition of information related to oral imaging diagnosis into the cases.
3. *Illustration from the oral pathologist (oral histopathology teacher)* First, the oral pathologist briefed the students on the preparation for and the surgical procedure, focusing on intraoperative frozen sections and postoperative routine histopathological sections. Next, according to the clinical characteristics and imaging diagnosis features, Case 1 was diagnosed preliminarily as an oral and maxillofacial cyst. Finally, the origin and biological characteristics of the cyst were analyzed under a microscope to distinguish it from an odontogenic cyst with poor biological characteristics.
4. *Simulation MDT groups imitated the above process* See Fig. 3 for details.

### The core ideas for cultivating the clinical thinking ability during the teaching process

1. To clarify contradictory and confirmatory relationships among clinical features, oral imaging, and histopathological diagnosis.
2. To explain the influence of oral pathology and oral imaging as diagnostic means on preoperative and intraoperative planning of an oral and maxillofacial surgical operation.
3. To explain the influence of MDT on the patient prognosis and surgical treatment of similar diseases in the future.
4. To teach humane care and doctor–patient communication skills.

### Satisfaction questionnaire

The satisfaction questionnaire included “cases teaching” and “paired teachers” patterns, and respectively existed in two questionnaires designed considering the necessity, predilection, degree of participation, mentality, and knowledge acquisition. There were a total of 10 items in two questionnaires and 3- or 4-point Likert scales, respectively. See Tables 1 and 2 for details.

**Table 1:** Survey on the recognition of Mimic MDT training based on case-teaching.

	Question	Answers	Frequency	Percent
1	“Do you like case teaching?	A. Very well	30	65.22
		B. Good	16	34.78
		C. Average	0	0
		D. Poor	0	0
2	Do you think it is necessary to enter a case teaching in oral histopathology?	A. Necessary	26	56.52
		B. No sense	15	32.61
		C. Not necessary	5	10.87
3	Do you be willing to involve a training for enhancing clinical thinking through case teaching,such as Mimic MDT training	A. Willing	36	78.26
		B. No sense	10	21.74
		C. Unwilling	0	0
4	Do you be anxious your performance in Mimic MDT training ?	A. No sense	5	10.87
		B. A little	31	67.39
		C. So much	10	21.74
5	Did you gain from participating in Mimic MDT training? What is the “gain”?	A. No sense	15	32.61
		B. Yes, Filling in the following column	30	65.22
		C. Almost nothing	1	2.17

**Table 2:** Survey on the recognition of Mimic MDT training based on paired-teachers.

	Qestions	Answers	Frequency	Percent (%)
1	“Do you like paired-teachers ?”	A. Very well	30	65.21
		B. Good	10	21.74
		C. Average	6	13.04
		D. Poor	0	0
2	Do you think it is necessary to apply a reform by the paired.-teachers way in oral histopathology?	A. Necessary	6	13.04
		B. No sense	35	76.09
		C. Not necessary	5	10.87
3	Do you be willing to involve a training for enhancing clinical thinking through the paired.-teachers way, such as Mimic MDT training	A. Willing	18	39.13
		B. No sense	25	54.35
		C. Unwilling	3	6.52
4	Do you think the Mimic MDT training by “paired.-teachers” guidance has advantages? What is that?	A. No sense	15	32.61
		B. Yes, Filling in the following column	25	54.35
		C. Almost nothing	6	13.04
5	Do you have any suggestions for the Mimic MDT training of “paired.-teachers” guidance?What is that?	A. No sense	16	34.78
		B. Yes, Filling in the following column	19	41.30
		C. Almost nothing	11	23.91

### Ethics approval and consent to participate

Ethical approval for this study was obtained from the Ethics Committee of the First Affiliated Hospital of Harbin Medical University. All methods were utilized in accordance with the relevant guidelines and regulations. Informed consent was obtained from all subjects involved in this study.

## Results

### Curriculum satisfaction survey

To further understand the effects of the simulation MDT training, we compiled a simulation-MDT-training satisfaction questionnaire and a paired-teacher-guided MDT satisfaction questionnaire. See Tables 1 and 2 for details.

The simulation MDT group agreed that the simulation MDT was novel for them and they had a strong sense of participation. As many as 54.35% of the interviewees thought that the advantage was that a typical case in this study was presented with sufficient information including clinical features, oral imaging diagnosis, and oral histopathological diagnosis. Multidisciplinary data including oral and maxillofacial surgery, oral imaging, and oral pathology were focused on a question or a case. There was a change in the thinking mode, from remembering trivial points on different subjects to the integration of details into a coherent concept depending on a case or problem at hand. More importantly, differential diagnosis of various cases often resulted in brainstorming, which was an enticing challenge, and the students examined potential diseases with similar characteristics. As many as 41.30% of all the participants hoped that the performance on the simulation MDT training would be included in the final grade (exam scores).

Nonetheless, 21.74% of the students felt substantial pressure because of the simulation MDT training; 76.09% were not sure whether the “Case + Teacher pairs + Simulation MDT” class should be offered in the future. Of note, 78.26% of the interviewees expressed their willingness to participate in simulation MDT activities. This seemingly contradictory result shows that the students did not pay attention to their own autonomy in reform teaching and passively accepted the arrangement handed down by the teachers.

## Discussion

MDT stands for a collaborative group for diagnosing and treatment of individual diseases in patients. This collaborative group provides a platform for the integration of a wide range of subspecialties, which represents the main direction of the future progress in personalized precision medical treatment and is the only way to train a medical elite. Therefore, the cultivation of MDT-type clinical thinking should be implemented gradually and carried out in the undergraduate training of medical students. In view of the above requirements, in this study, we conducted mimic MDT training based on typical cases with guidance from oral imaging and oral pathology teachers for undergraduates in a 5-year stomatology program in their junior-year second semester.

### The combination of a “Case” with “Teacher pairs” as a good approach to simulation multidisciplinary diagnosis and treatment

The key points of mimic MDT are the selection of typical cases and coordination by the instructor. The students can be directed for the cultivation of clinical thinking in the process of simulation MDT. That is, they can “zoom in” on a subspecialty and “zoom out” to the multidisciplinary mode through typical cases.

In this study, we selected the cases where the hints offered in imaging diagnosis were “contradictory to,” “inconsistent with,” or “irrelevant for” the final histopathological diagnosis, and these cases aroused much interest among the students. The causes of “contradiction,” “disunity,” and “irrelevance” were revealed through preoperative examination, intraoperative frozen sections, and postoperative routine histopathological examination results, which triggered brainstorming among the students. Next, the students clarified the advantages and limitations of each medical test. Comprehensive application of various medical tests is an essential ability in future learning, and the final diagnosis is a process of complementation and verification by various clinical investigative methods.

### Simulation MDT inspires students’ and teachers’ learning and exploration

The questionnaires showed that students have a deeper understanding of learning materials because of the simulation MDT training. Clinical manifestations, imaging diagnosis, and the formulation of a final treatment plan may vary, although final histopathological diagnoses of individual diseases may be identical. This study found that mimic MDT can effectively train students to use divergent thinking, lay the foundation of diagnosis basics, and improve the capacity for differential diagnosis. Nonetheless, these reforms put a strain on those students who are good at rote learning which is a memorization technique based on repetition on college exams. Student difficulties in this setting may be caused by inertia, that is., the continuation of the previous learning mode (which is suitable for college entrance examinations in China) according to interviews and questionnaire results. Of note, although students were curious about the attempted teaching reform, they did not show initiative in this process. Furthermore, a few students were resistant to some types of challenging independent learning. We think that the inertia, that is, the continuation of the previous study mode (examination-oriented learning) and indolent self-directed learning both need to be corrected and not encouraged among students, although we are risking a low course satisfaction rating.

It is worth noting that some students suggested that MDT course scores should be included in the final examination scores. We also believe that a comprehensive assessment of thinking ability should be a part of student ability training. Nevertheless, this process needs more research data support, and it is also necessary to re-analyze, refine, and adjust the syllabus under this teaching model.

However, we found that the knowledge structure of the participating teachers also changed and become more systematic and logical in the course of preparing and teaching, which would convince them to adopt to some degree those new educational approaches. Meanwhile, school administrators are encouraging educational reform and providing facilities to match it, which contributes to increasing the level of teacher involvement.

The combination of case-based learning and MDT has also been reported in stomatology education in China. Nevertheless, these are based on a single disease or a subject, such as temporomandibular joint diseases^16^, oral cancer^17^, and periodontology^18^. We focused on a group of diseases with similar clinical manifestations but completely different in definitive diagnosis, which includes cysts, tumors, tumor-like lesions, and non-neoplastic diseases. Students would face a greater challenge. Furthermore, we explored the role of diagnosis means with subjectivity including oral imaging and oral pathology. “Inconsistent details” or “mutual corroboration details” between them are useful in shaping and improving student clinical thinking. Unfortunately, we currently have no means to quantify clinical thinking changes and can only describe variation through the subjective feelings of students. Interestingly, Richards et al. found that interdisciplinary, case-based teaching increased students' appreciation of the complexity of patient care and a patient-centered, culturally sensitive approach to diagnosis and treatment planning^19^.

### Questions and limitations

The key points are a proper case illustration process and logical guidance of clinical thinking from teachers. Simultaneously, students need to have some basic knowledge. For example, in this study, all students who participated in this class activity completed the coursework on pathology, diagnostics, imaging, and surgery in their sophomore year and the first semester of their junior year. Continuation of the previous study mode (examination-oriented learning) and indolent self-directed learning may be negative factors for simulation MDT training.

The number of students participating in this study was limited, and more students are should participate next year. The number of cases was gradually increased for better consolidated clinical thinking. These cases may form a network that can cover wider knowledge points of the syllabus.

## Conclusions

The mimic MDT training with typical cases guided by paired teachers was useful for establishing student clinical thinking. The higher participation and better satisfaction of the students in this innovative course are a considerable achievement and serve as a good precedent for the cultivation of student clinical thinking during early basic stomatology education.

## Data Availability

The data are kept at Department of Oral Pathology, Hospital of Stomatology, the First Affiliated Hospital, Harbin Medical University, Harbin 150001, P. R. China. Any questions or requests regarding the data can be addressed to Yanfen Qiu (1679314251@qq.com).
